# SSC-ILD mouse model induced by osmotic minipump delivered bleomycin: effect of Nintedanib

**DOI:** 10.1038/s41598-021-97728-z

**Published:** 2021-09-16

**Authors:** Francesca Ravanetti, Erica Ferrini, Luisa Ragionieri, Zahra Khalajzeyqami, Maria Nicastro, Yanto Ridwan, Alex Kleinjan, Gino Villetti, Andrea Grandi, Franco Fabio Stellari

**Affiliations:** 1 Department of Veterinary Science, University of Parma, Parma, Italy; 2 Department of Veterinary Medical Sciences, University of Bologna, Bologna, Italy; 3 Department of Molecular Genetics, Vascular Surgery and Radiation Oncology, Erasmus MC, Rotterdam, The Netherlands; 4 Department of Pulmonary Medicine, Erasmus MC, Rotterdam, The Netherlands; 5 Pharmacology & Toxicology Department, Chiesi Farmaceutici S.p.A., Corporate Pre-Clinical R&D, Largo Belloli, 11/A, 43122 Parma, Italy

**Keywords:** Animal disease models, Respiratory system models, Skin models

## Abstract

Systemic sclerosis (SSc) is an autoimmune disease characterized by an excessive production and accumulation of collagen in the skin and internal organs often associated with interstitial lung disease (ILD). Its pathogenetic mechanisms are unknown and the lack of animal models mimicking the features of the human disease is creating a gap between the selection of anti-fibrotic drug candidates and effective therapies. In this work, we intended to pharmacologically validate a SSc-ILD model based on 1 week infusion of bleomycin (BLM) by osmotic minipumps in C57/BL6 mice, since it will serve as a tool for secondary drug screening. Nintedanib (NINT) has been used as a reference compound to investigate antifibrotic activity either for lung or skin fibrosis. Longitudinal Micro-CT analysis highlighted a significant slowdown in lung fibrosis progression after NINT treatment, which was confirmed by histology. However, no significant effect was observed on lung hydroxyproline content, inflammatory infiltrate and skin lipoatrophy. The modest pharmacological effect reported here could reflect the clinical outcome, highlighting the reliability of this model to better profile potential clinical drug candidates. The integrative approach presented herein, which combines longitudinal assessments with endpoint analyses, could be harnessed in drug discovery to generate more reliable, reproducible and robust readouts.

## Introduction

Systemic sclerosis (or scleroderma) (SSc) is an autoimmune disease of unknown aetiology, characterized by vasculopathy, excessive fibrous tissue generation and aberrant immune activation resulting in damage to various organs including the skin, esophagus, heart, lungs, and kidneys^1,2^. Most critically, about 80% of SSc patients develop pulmonary dysfunctions such as pulmonary hypertension and associated interstitial lung disease (ILD), this latter characterized by early immune cell infiltration followed by various degrees of fibrosis and gas exchange impairment that significantly reduce life expectancy^3–5^. The pronounced uncertainty surrounding the pathogenetic mechanisms behind SSc-ILD and the absence of effective treatments for this disorder have solicited the creation of ad hoc animal experimental models capable of mimicking the different clinical and pathological peculiarities of SSc-ILD in humans^6–8^.

Bleomycin (BLM) is the most commonly used agent to replicate some ILD key hallmarks, such as changes in pulmonary histoarchitecture, fibroblast/myofibroblast activation, and collagen deposition^9–11^. Over the past few years, many studies have tested various methods of BLM administration^12–14^. In particular, its systemic delivery through subcutaneously implanted osmotic minipumps in mice has been used to experimentally mimic the typical features of systemic sclerosis and related interstitial lung diseases^14,15^.

Before using a new animal model in drug discovery, its reproducibility, robustness and ability to respond to pharmacological treatments should be validated. The selection of the best drug candidate to be introduced into the clinic depends on many factors, but a head-to-head comparison with a gold standard therapy has an important role. Several crucial points, such as time, doses of administration and different readouts, are essential to provide the best view on how drugs are performing in animal models, since each model can respond to the same therapy in a different manner.

A model inducing subacute—chronic fibrosis through the release of BLM by osmotic mini-pumps allows evaluation about the effects of antifibrotic compounds, either in the skin or in the lungs, since the target fibrotic lesions have been identified in both organs^15–17^. The main purpose of this study was to examine the antifibrotic activity of nintedanib (NINT), a tyrosine-kinase inhibitor used to treat idiopathic pulmonary fibrosis and recently approved as a therapy for SSc associated with ILD^18,19^, in the aforementioned SSc-ILD mouse model.

Recent results of clinical trials showed that NINT has a beneficial effect reducing the rate of decline in forced vital capacity in patients with ILD associated with SSc over 1-year period, even though no clinical benefits of NINT was observed for other manifestations of SSc^18,19^. As regard to animal models, the in vivo efficacy of NINT was explored either on silica-induced lung fibrosis in mice or BLM-induced lung fibrosis in mice and rats^20–23^, but it has not yet been tested in the SSc-ILD mouse model using BLM delivered through osmotic minipumps. In the present research, NINT was orally administered for 2 weeks following a therapeutic protocol after acute inflammation phase, starting at day 14^24,25^. In order to use this fibrosis model for secondary screening in our pipeline several readouts were considered. The lung fibrosis progression and the pharmacological response to treatment were longitudinally assessed by micro-CT in the same mice and then corroborated by histological analyses in lung and skin samples at the experimental endpoint.

## Materials and methods

### Animal model

Twenty-four 7–8-week-old C57BL/6 female mice (Envigo, San Pietro al Natisone, Udine, Italy) were kept in a conventional animal facility in ventilated cages with free access to standard rodent chow and softened tap water at least 7 days prior to use. All mice were randomly subdivided into two groups: 8 were treated with saline and 16 with BLM. All experiments were carried out in accordance with the intramural animal-welfare practices for animal experimentation of Chiesi Farmaceutici and complied with the European Directive 2010/ 63 UE, Italian D.Lgs 26/2014 as well as the revised “Guide for the Care and Use of Laboratory Animals” (National Research Council Committee, US, 2011)^26^ and ARRIVE guidelines^27^. The experimental protocol was approved by the internal AWB (Animal Welfare Body) and authorized by Italian Ministry of Health *(protocol number: 449/2016-PR).*

### Experimental design

**Figure 1:**
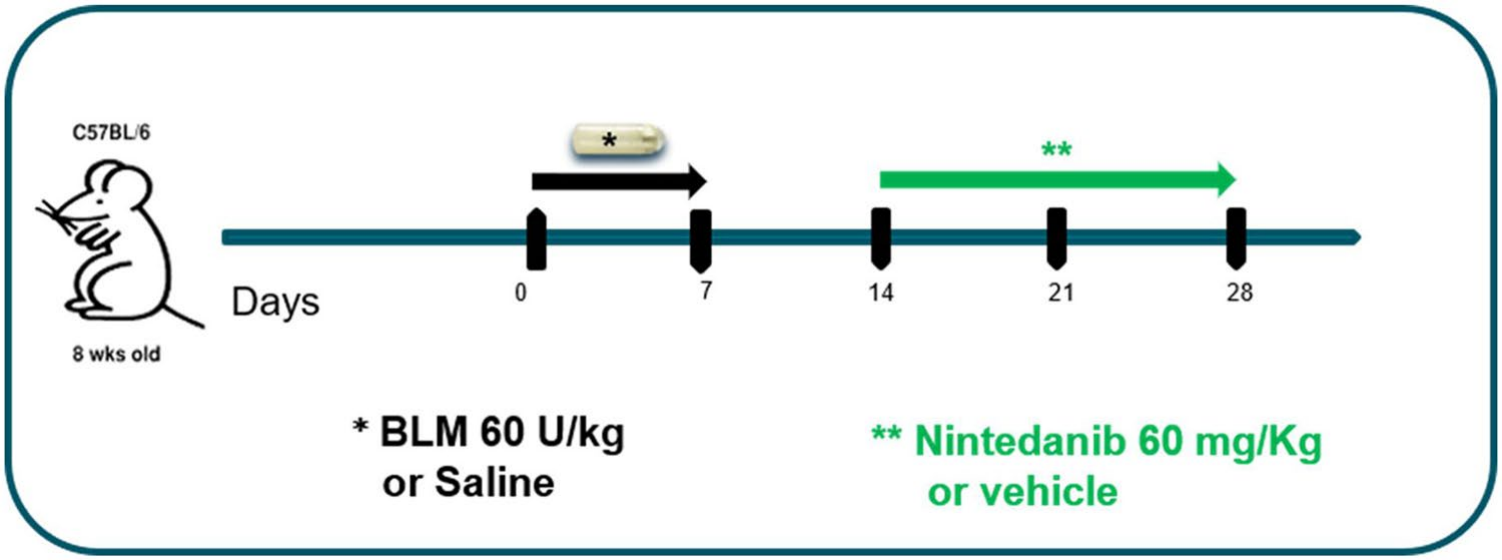
Experimental set up. Experimental timeline of bleomycin-induced lung mouse fibrosis by osmotic minipumps. Twenty-four females C57BL/6 were randomly assigned to receive either saline (8 mice) or BLM (60U/kg) by osmotic minipumps at day 0. From day 14, 8 animals from BLM group received nintedanib and the others 8 received vehicle until day 28. At the endpoint, the effect of nintedanib was assessed by micro-CT and ex-vivo analyses.

Each mouse was anesthetized with 2.5% isoflurane mixed with oxygen, the implantation site was shaved and, through a small incision, a subcutaneous pocket was created on the left-hand side of its back using the jaws of a hemostat clamp. The osmotic minipumps [ALZET 1007D; DURECT, (release rate 0.5 µl/h for 7 days), Cupertino, California, US] containing either 100 µl saline or BLM (60 U/kg dissolved in saline, Baxter Oncology GmbH, Halle (Westf.), Nordrhein-Westfalen, Germany ) were implanted and removed after 8 days. At day 14, BLM mice were randomly divided in two groups, receiving either NINT (60 mg/kg/day, Carbosynth Limited, Compton, UK) dissolved in Tween80 0.05% in saline or vehicle (Tween80 0.05% in saline), by gavage, daily for 2 weeks (Fig. 1).

The animals were monitored and weighed daily throughout the experimental procedure.

### Micro-computed tomography acquisition protocol

Micro-computed tomography (micro-CT) lung imaging was performed longitudinally at day 14 and 28 by Quantum GX Micro-CT (PerkinElmer Inc., Waltham, Massachusetts, US). Each mouse was anesthetized using 2% isoflurane and then positioned inside the CT scan. Images were acquired with the following parameters: X-ray tube voltage 90 kV, X-ray tube current 88 µA and total scan time of 4 min. A ring reduction correction was applied to the sinograms and the entire set of projection radiographs was entered into a GPU-based filtered back-projection algorithm with a Ram-Lak filter^28^. The acquisition protocol in ‘high resolution’ mode resulted in one 3D dataset with 50 μm isotropic reconstructed voxel size.

### Image post‑processing: lung segmentation protocols and analysis

For each acquisition, a stack of 512 cross-sectional images was produced. The reconstructed datasets were analyzed using the Perkin Elmer Analyze software (Analyze 12.0; Copyright 1986–2017, Biomedical Imaging Resource, Mayo Clinic, Rochester, Minnesota, US). The images stacks were filtered and converted from grey levels to CT numbers (Hounsfield Units—HU). The conversion is a linear transformation setting −1000 HU as the density of air and 0 HU as the density of water. A semi-automatic segmentation was used to extract airways and lungs. For the quantitative assessment of the lung parenchyma, HU clinical ranges were applied on rescaled HU images to the segmented lung volume to define normo-aerated [(−900, −500) HU] and poorly-aerated [(−500, −100) HU] tissues^29^. The two compartments with a different aeration degree were expressed as percentage of the total lung volume. The poorly-aerated tissue refers to a low gas/tissue ratio and it was used to quantify lung fibrosis progression and evaluate the efficacy of NINT^30^.

### Bronchoalveolar lavage, cytokines, and matrix metalloproteinases

After micro-CT imaging at day 28, all mice were euthanized with an overdose of anesthetic follow by bleeding from the abdominal aorta. Bronchoalveolar lavage fluid (BALF) was collected by gently washing the bronchial tree using 0.6 mL sterile solution three times [Hank’s balanced salt solution (HBSS) 1×; ethylenediaminetetraacetic acid (EDTA) 10 mM; 4-(2-hydroxy-ethyl)-1-piperazineethansulphonic acid (HEPES) 10 mM].

The samples were centrifuged at 300×*g* for 10 min at 4 °C and the supernatant collected and frozen for further investigation. The cellular pellet was resuspended in 0.2 mL of BALF solution and total white blood cells (WBC) were measured using an automated cell counter (Dasit XT 1800 J). Afterwards, about 1.0 × 10^6^ cells were also used to quantify the M2 macrophage population by flow cytometry. The cells were suspended in FACS Buffer (PBS; 0.5% BSA) and in the lysis buffer to remove red blood cells (BD Bioscience). Then the cells were stained with anti-CD206 (Bio-Rad), anti-F4/80 (BioLegend) and anti-CD11b (BioLegend) antibodies, washed, and finally acquired using a FACS Canto II Cytometer (BD Bioscience) and analyzed with FACS Diva software. The total macrophage population was selected based on forward (FSC) versus side scatter (SSC) plots, and, subsequently, M2 macrophages were identified in terms of total number of CD206^+^ events within a F4/80^+^CD11b^+^ selected population.

The matrix metalloproteinases 2 and 9 (MMP-2 and MMP-9, respectively) and metallopeptidase inhibitor 1 (TIMP-1) concentrations in BALF were assessed by enzyme-linked immunosorbent assay (ELISA) kit (R&D Systems, Minneapolis, USA). The protein concentrations were measured by interpolation from the standard curve and were expressed in fold of increase (FOI).

### Histological analysis and fibrosis quantification

After micro-CT and BALF analysis, at day 28, the whole left lung and skin from the left gluteal region (*i.e.,* distant from the implant site to avoid dermal fibrosis artifact) were excised. The skin and the lung were fixed with 10% neutral-buffered formalin and fixed for 24 h and embedded in paraffin. Serial 5 μm thick sagittal sections were stained with hematoxylin–eosin (H&E) to demonstrate the general tissue morphology, and with Masson’s trichrome (MT) to evaluate the collagen-based matrix. Whole slide images (WSI) were acquired using NanoZoomer S60 scanner (Hamamatsu Photonics K.K., Hamamatsu City, Japan). Two independent researchers with experience in animal models of lung fibrosis performed blind histological analyses.

**Figure 2:**
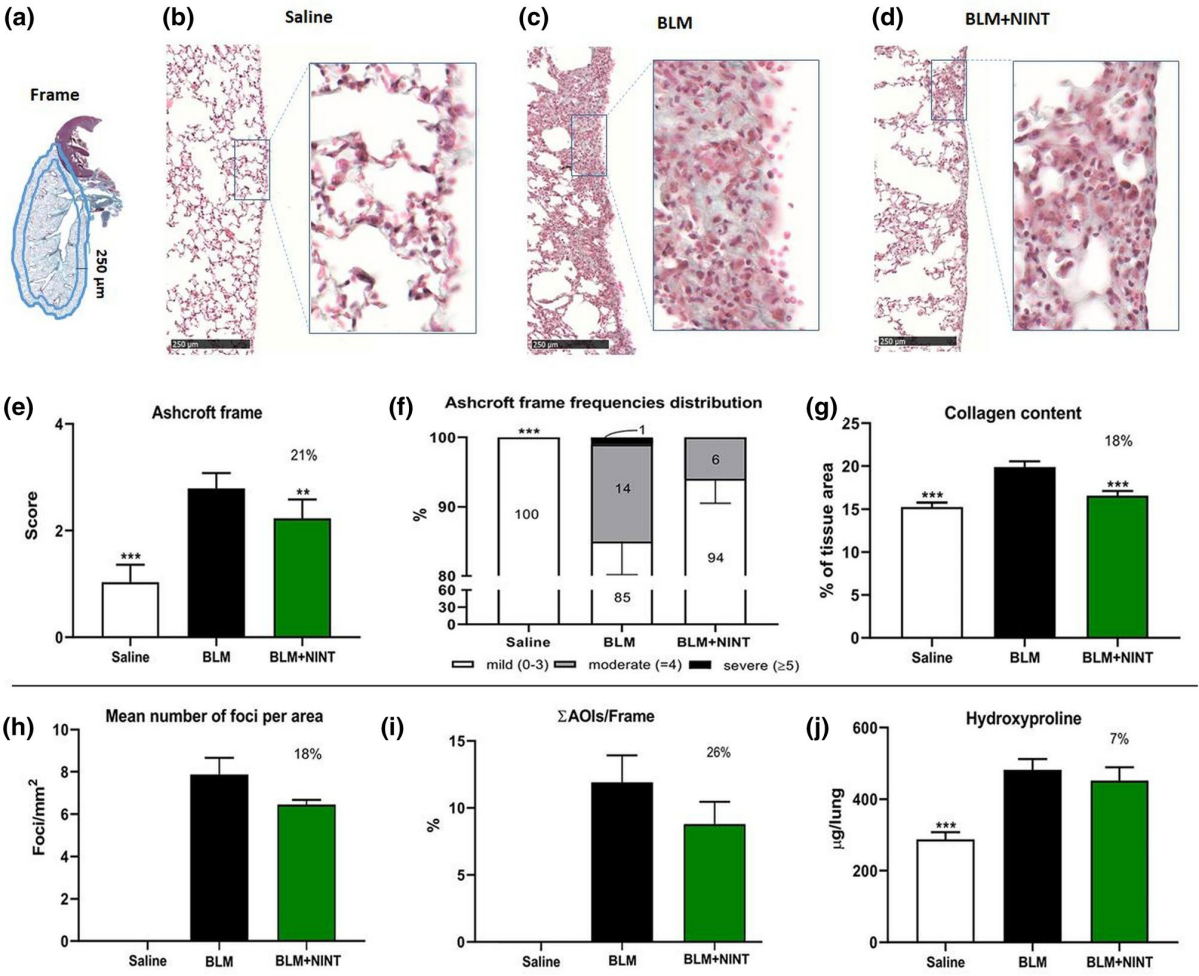
Histological and histomorphometric analysis of the subpleural parenchyma. Evaluation of the antifibrotic activity of nintedanib (on bleomycin induced lung fibrosis through osmotic minipumps) at day 28. (**a**) Schematic representation of the Frame 250-µm thick considered as a region of interest. (**b-d**) Representative microphotographs of the subpleural parenchyma of Saline (**b**), BLM (**c**) and BLM + NINT (**d**) treated groups at day 28 (MT staining; ×10 magnification. The area within the rectangles has been magnified at ×20). (**e**) Ashcroft score determination on the frame. (**f**) Frequency distribution (%) of the Ashcroft scores values grouped as mild (0–3), moderate (4) and severe (≥ 5). (**g**) Collagen content determination expressed as a percentage of the tissue area. **h**: Mean number of fibrotic foci per mm^2^ of Frame. (**j**) Global AOI area (AOIs) normalized on the Frame surface (AOIs/Frame) and expressed as percentage. **i:** Anti-fibrotic effect of NINT on hydroxyproline concentration in the right lung. Data are shown as mean ± S.E.M. for 8 mice per group. In **d, f, g, h** and **i,** Saline and BLM + NINT were compared to the BLM group using one-way ANOVA followed by Dunnett’s test (**P < 0.01; ***P < 0.001). In **e** statistical comparisons with the BLM group were performed using Chi-squared test (**P < 0.01; ***P < 0.001). The percentages on the BLM + NINT bars represent the inhibition effect of the treatment compared to BLM.

The extent of fibrosis was morphologically and qualitatively assessed in a subpleural frame (Region of Interest) of lung parenchyma (250 µm thickness, Fig. 2a). The fibroproliferative modifications in the frame area were evaluated through the semiquantitative 0-to-8 Ashcroft score. The Ashcroft frame scores were subsequently categorized into mild (mean score from 0 to 3), moderate (4) and severe (5–8). Moreover, the fibrotic foci within the frame were quantified based on morphological and colorimetric thresholds and labelled as “areas of interest” (AOI). The extent of fibrosis was evaluated through the following histomorphometric parameters: (1) mean number of fibrotic foci per mm^2^ of parenchyma; (2) focus size (small, if its area was < 7,500 mm^2^; large, if > 7,500 mm^2^); (3) fraction of Frame occupied by total AOI area (ΣAOI area/Frame surface), as a percentage value.

For the skin samples, the histomorphometric parameters considered in the MT stained sections were: (1) dermis thickness, defined as the mean distance between the epidermal-dermal junction and the dermal-subcutaneous junction^16^; (2) hypodermis thickness, defined as the mean distance between the dermal-subcutaneous junction and the muscle layer. The inflammatory infiltrate was evaluated on sections stained with H&E by a semiquantitative method using a 0 to 4 grade score, reflecting increasing inflammation, as described by Gallet et al. (2011)^31^. Measurements were carried out in five randomly selected fields from one sample from each animal.

### Collagen content

The collagen-based extracellular matrix was measured using the image analysis software NIS-Elements AR 3.1 (Nikon, Tokyo, Japan) in the TM-stained lung sections after selection of a correct green threshold detected on the Light Green stained collagen fibers to eliminate air spaces and bronchial epithelium^32^.

### Hydroxyproline quantification

The right lung lobes were used to quantify the collagen indirectly through hydroxyproline (Hyp) concentration using a commercial kit (Sigma-Aldrich) in accordance with the manufacturer’s protocol. In brief, the lobes were homogenized in PBS, hydrolyzed in 6 N HCl for 24 h at 100 °C and finally neutralized in 6 N NaOH. The final Hyp concentration was determined by the reaction of oxidized Hyp with 4-Dimethylamino benzaldehyde (DMAB), which resulted in a colorimetric product, proportional to the Hyp content. This reaction was measured at a wavelength of 560 nm and, finally, each total amount of Hyp was normalized for the relevant right lobe weight.

### Immunofluorescence staining

Immunofluorescent (IF) reactions were performed on paraffin embedded sections to detect M2-like polarized macrophages in the lung and Collagen type I in the skin. Briefly, paraffin embedded sections were deparaffinized, rehydrated, and then antigen retried (in a 10 mM citrate buffer at a boiling point). After a cooling step, the slides were rinsed in a wash buffer and then immersed in a blocking buffer (0.3 M glycine, 5% bovine serum albumin in 1_ PBS; Sigma-Aldrich, St Louis, Missouri, US) at room temperature. Sections were incubated using primary antibody (anti-CD206: 1 µg/mL, AF2535; R&D Systems—anti-Collagen type I: 5 µg/mL, ab88147; Abcam, Cambridge, UK)). This reaction was revealed by a specific secondary antibody (donkey anti-goat antibody Alexa Fluor 488 conjugate: 3 µg/mL, AB2336933; Jackson Laboratories, Bar Harbor, Maine, US—Goat Anti-Mouse IgG, Fcγ subclass 3 specific Rhodamine Red™-X (RRX) AffiniPure (dil. 1:200; 115-295-209; Jackson ImmunoResearch, Ely, Cambridgeshire, UK). Lastly, the nuclei were counterstained with DAPI (Invitrogen). For negative control the primary antibody was omitted and tissues were incubated in 10 mM phosphate buffer or, alternatively, with unlabelled rabbit IgG nonimmune isotype control (2009–1; Alpha Diagnostic International) used at the same concentration as the selective antibody. Fluorescent WSI were acquired using NanoZoomer S60 (Hamamatsu Photonics K.K., Hamamatsu City, Japan).

### Statistical analysis

Statistical analysis was performed using one-way ANOVA followed by Dunnett’s *t* test, to compare each group with the BLM group as control. For micro-CT data a two-way ANOVA test was performed to compare each group with the BLM group and to compare different time points of observation, using Dunnett’s and Sidak’s tests for multiple comparisons, respectively. The comparison of the frequency distribution was performed with a Chi-squared test. Statistics were carried out using GraphPad Prism 7.0 software (GraphPad; La Jolla, California, US). Sample size was calculated with A-priori Power Analysis (G*Power Version 3.1.2) considering Ashcroft Score as endpoint. A value of *P* < 0.05 was considered statistically significant. Data are expressed as mean ± S.E.M.

## Results

### Experimental animals

BLM induced a weight loss up to a maximum reduction of 18% at day 14 but, as expected, mice recovered at later time points.

NINT showed a well-tolerated profile, since no difference in body weight was identified compared to the BLM group. The Saline group did not exhibit any distress and no weight loss was observed (Supplementary Fig. 1).

### Morphology and fibrosis quantification in subpleural tissue

MT staining highlighted different morphological features in the lungs of BLM and BLM + NINT groups, as compared to the normal parenchyma of the Saline group. Representative foci histological features and conglomerations are presented in Fig. 2(b-d). The fibrotic lesions were mainly located in the subpleural lung parenchyma (frame) in both BLM and BLM + NINT groups and were characterized by collagen deposition with thickening alveolar septa and moderate inflammatory infiltrate.

BLM induced a significant increase in Ashcroft score compared to Saline, while NINT treatment significantly mitigated lung fibrosis, showing a 21% reduction with respect to BLM (Fig. 2e).

The frequency distribution of Ashcroft score values showed predominantly mild (85%) and moderate (14%) lesions in BLM group. The antifibrotic treatment modulated the fibrosis, increasing the percentage of mild (94%) and decreasing the moderate (6%) fibrotic lesions (Fig. 2f).

In accordance with the Ashcroft score, collagen content percentage was significantly reduced in NINT-treated mice compared to the BLM group (18%) (Fig. 2g).

The effect of NINT was also investigated for number of foci/mm^2^ and ΣAOI area/Frame (%). This analysis highlighted significant differences between the Saline and BLM groups, but was not able to discriminate the efficacy of the antifibrotic therapy, since both these parameters were not significantly reduced by the NINT treatment (Fig. 2h, j). Similarly, the quantification of Hyp in the lungs of the BLM group was significantly higher compared to Saline but not significantly reduced by NINT treatment (Fig. 2i).

**Figure 3:**
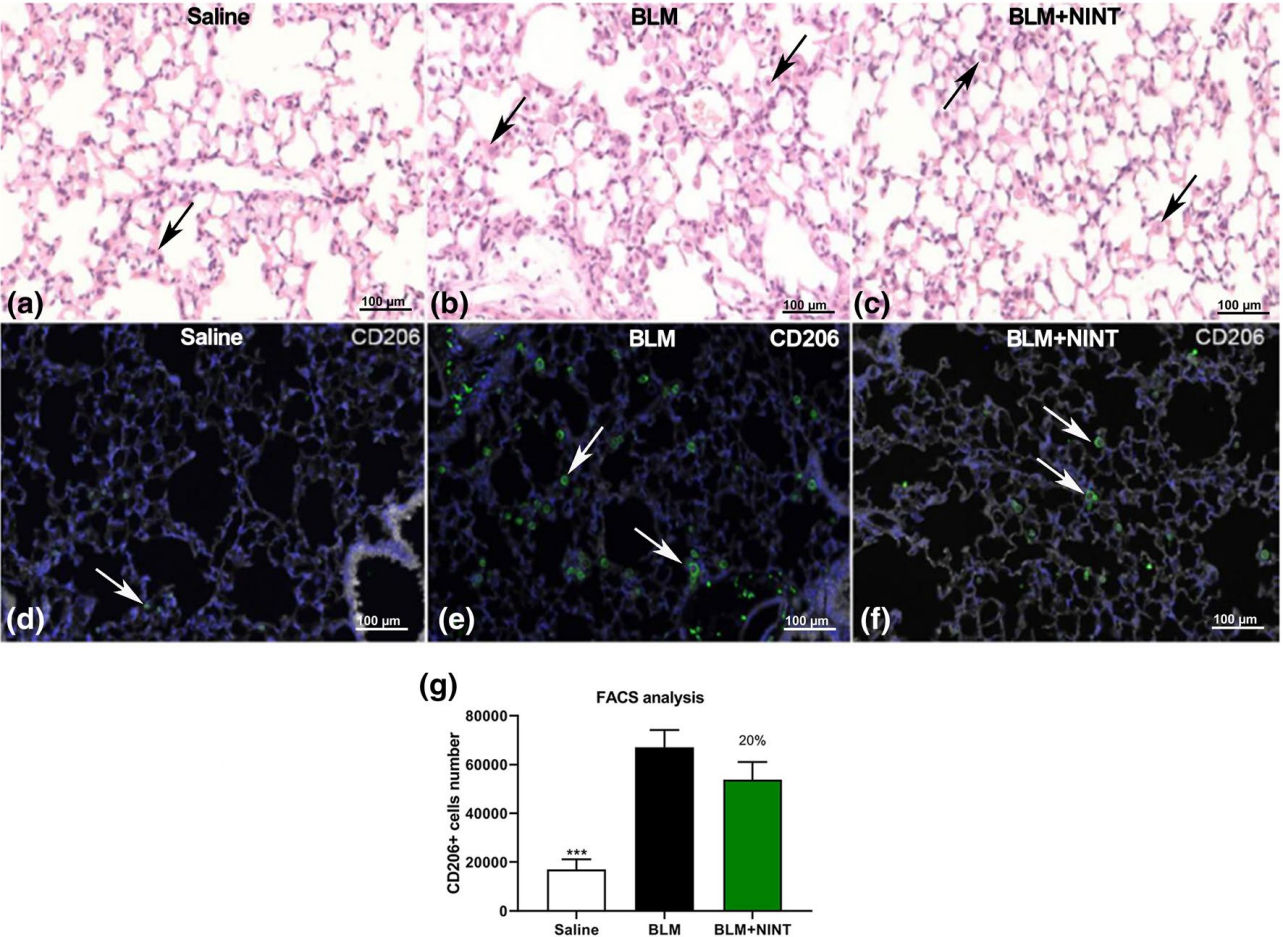
Detection of macrophages in the lung tissue and BALF. Representative microphotographs of sequential sections of lung tissue of saline (**a** and **d**), BLM, (**b** and **e**) and BLM + NINT treated mice (**c** and **f**) at day 28. (**a**, **b**, **c**: H&E staining; **d**, **e**, **f**: immunofluorescent staining for CD206 and DAPI). Residual macrophage cells in lung tissue after bronchoalveolar lavage are indicated with black arrowheads in the H&E images. White arrowheads indicate macrophages expressing CD206^+^ in IF images. (**g**) Absolute number of CD206^+^ macrophages counted by FACS in saline, BLM and BLM + NINT-treated mice. Data are shown as mean values ± S.E.M. for 8 mice per group. Asterisks indicate significant statistical differences in comparison with BLM group (***P < 0.001; ANOVA followed by Dunnett’s test). The percentage on the BLM + NINT bars represents the inhibition effect of the treatment compared to BLM.

BLM induced pulmonary inflammation by recruiting white blood cells (WBC), (Supplementary Fig. 2a). NINT treatment significantly reduced (40%) the total number of WBC measured in BALF, but only a modest inhibition was observed on the macrophage (26%), lymphocyte (24%) and neutrophil (33%) populations (Supplementary Fig. 2a–d). We have investigated the effect of NINT on the M2-like macrophages cell population in BALF, using a flow cytometry technique with CD206 as a surface marker. FACS analysis revealed a marked increase in M2-like cells in BLM-treated mice, compared to Saline, however no significant level of inhibition was observed in the BLM + NINT group (Fig. 3g).

IF staining of lung tissue after bronchoalveolar lavage confirmed the presence of residual M2-like macrophages in both BLM and BLM + NINT-treated mice (Fig. 3a–f).

**Figure 4:**
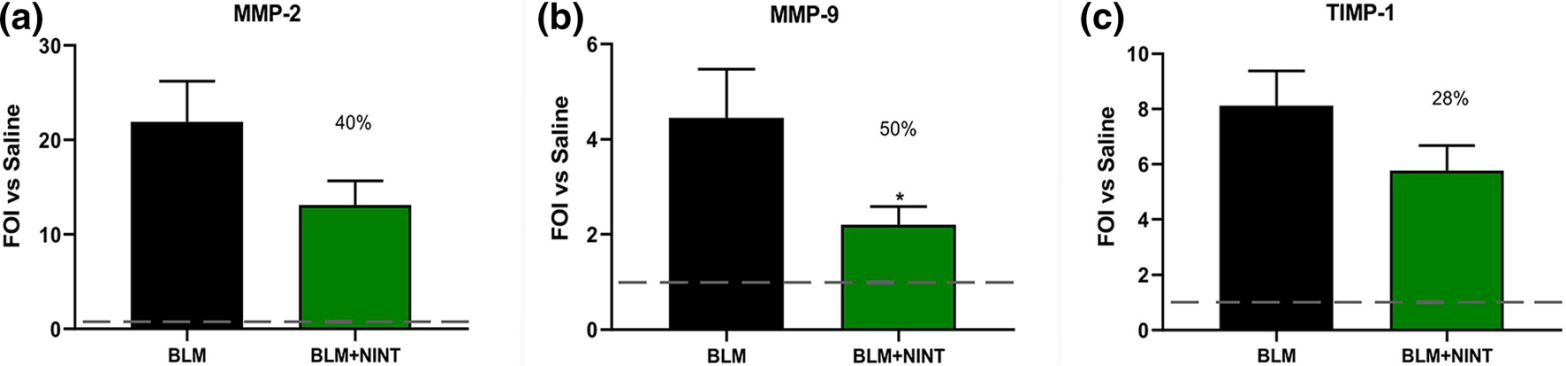
Evaluation of antifibrotic activity of nintedanib on matrix metalloproteinases at day 28 in BALF. MMP-2 (**a**), MMP-9 (**b**) and their inhibitor TIMP-1 (**c**) measured using the specific ELISA kit. The fold of increase vs Saline (FOI vs. Saline) for each group is shown as mean ± S.E.M. for 8 mice per group. The dashed lines indicate the saline values. Asterisks indicate statistical significance of each group vs. BLM (*P < 0.05; one-way ANOVA followed by Dunnett’s test). The percentages on the BLM + NINT bars represent the inhibition effect of the treatment compared to BLM.

Antifibrotic treatment inhibited MMP-2 (40%), MMP-9 (50%) and TIMP-1 (28%) concentrations in BALF, however, statistical significance was reached only for MMP-9 (Fig. 4a–c).

**Figure 5:**
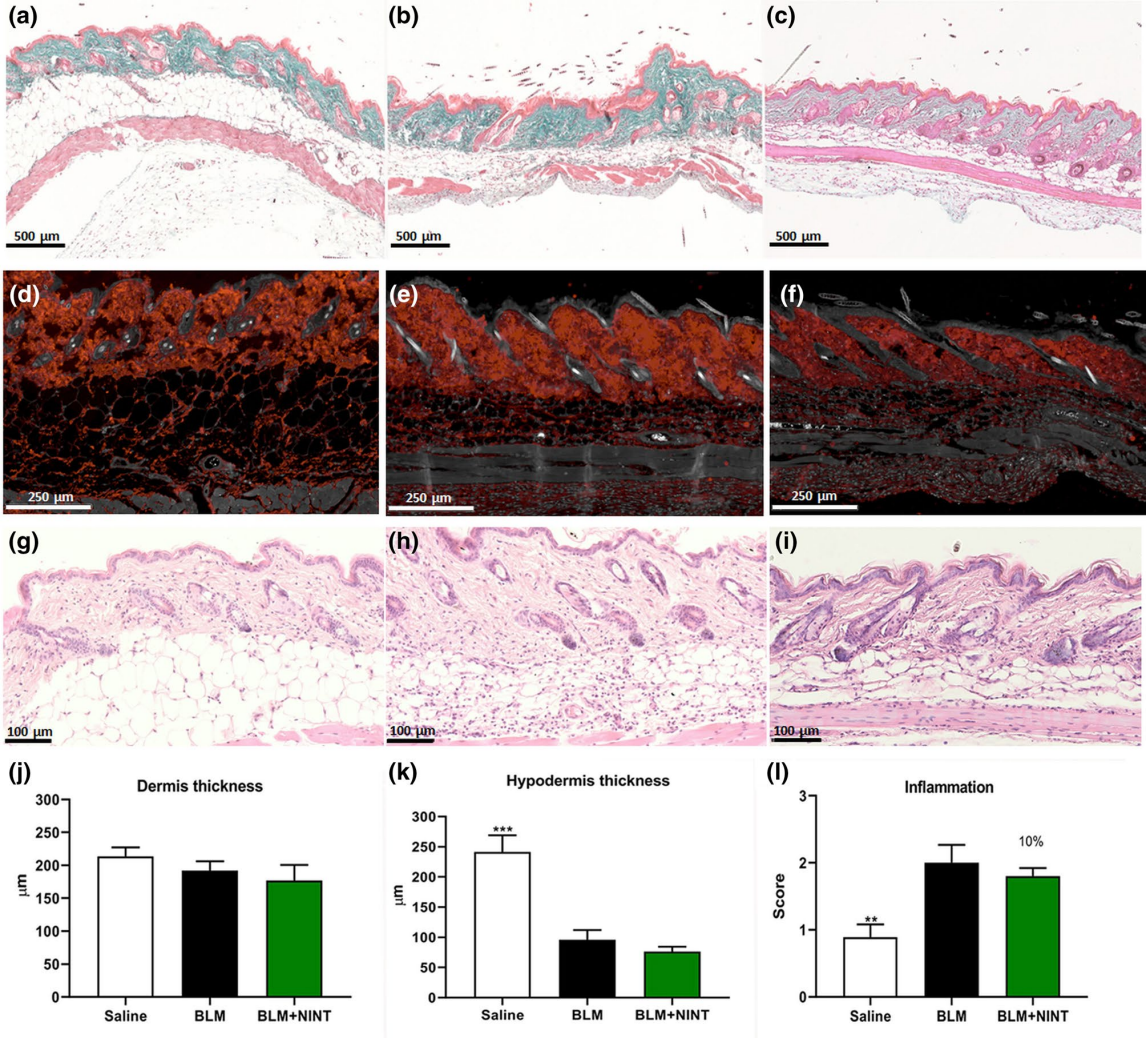
Histomorphometric analysis of skin fibrosis. Evaluation of the antifibrotic activity of nintedanib on bleomycin-induced skin fibrosis through osmotic minipumps at day 28. Skin microphotographs of Saline (**a, d, g**), BLM (**b, e, h**) and BLM + NINT (**c, f, i**) groups. MT stained images (**a, b, c**—× 5) showing differences in hypodermis thickness between control and treated animals. Immunofluorescent staining for Collagen Type I (**d, e, f**—× 15) showing different levels of expression in control and treated animals. H&E staining (**g, h, i**—× 30) showing different inflammatory infiltrates in control and treated animals. Measures of dermis thickness (**j**), hypodermis thickness (**k**) and evaluation of the inflammation score (**i**) were carried out at five randomly selected fields from a sample of each animal. Data are shown as mean values ± S.E.M. for 8 mice per group. Changes were compared to the BLM group using one-way ANOVA followed by Dunnett’s test (**P < 0.01; ***P < 0.001). The percentages on the BLM + NINT bars represent the inhibitory effect of the treatment compared to BLM.

Another goal of this study was to evaluate the activity of NINT treatment on skin fibrosis. The most evident changes in skin morphology of BLM and BLM + NINT, compared to Saline, are due to lipoatrophy (Fig. 5a–c). Regarding the distribution of collagen fibers, their compactness increased in BLM treated animals as compared to Saline, both in loose and dense irregular connective tissue of the dermis and in reticular connective tissue of the hypodermis layer (Fig. 5a–c; d–f). No significant differences were found in dermis thickness (Fig. 5j), while a significant decrease in hypodermis thickness was observed in both BLM and BLM + NINT compared to Saline-treated mice (Fig. 5k). Finally, a moderate pro-inflammatory effect of BLM was revealed in skin as documented by the inflammatory infiltrate (Fig. 5g, h, i) and only a modest decrease of the inflammatory score was induced by NINT (Fig. 5l).

### Micro-CT

Longitudinal micro-CT imaging was performed at 14 (baseline) and 28 days, representing the beginning and the end of NINT treatment.

**Figure 6:**
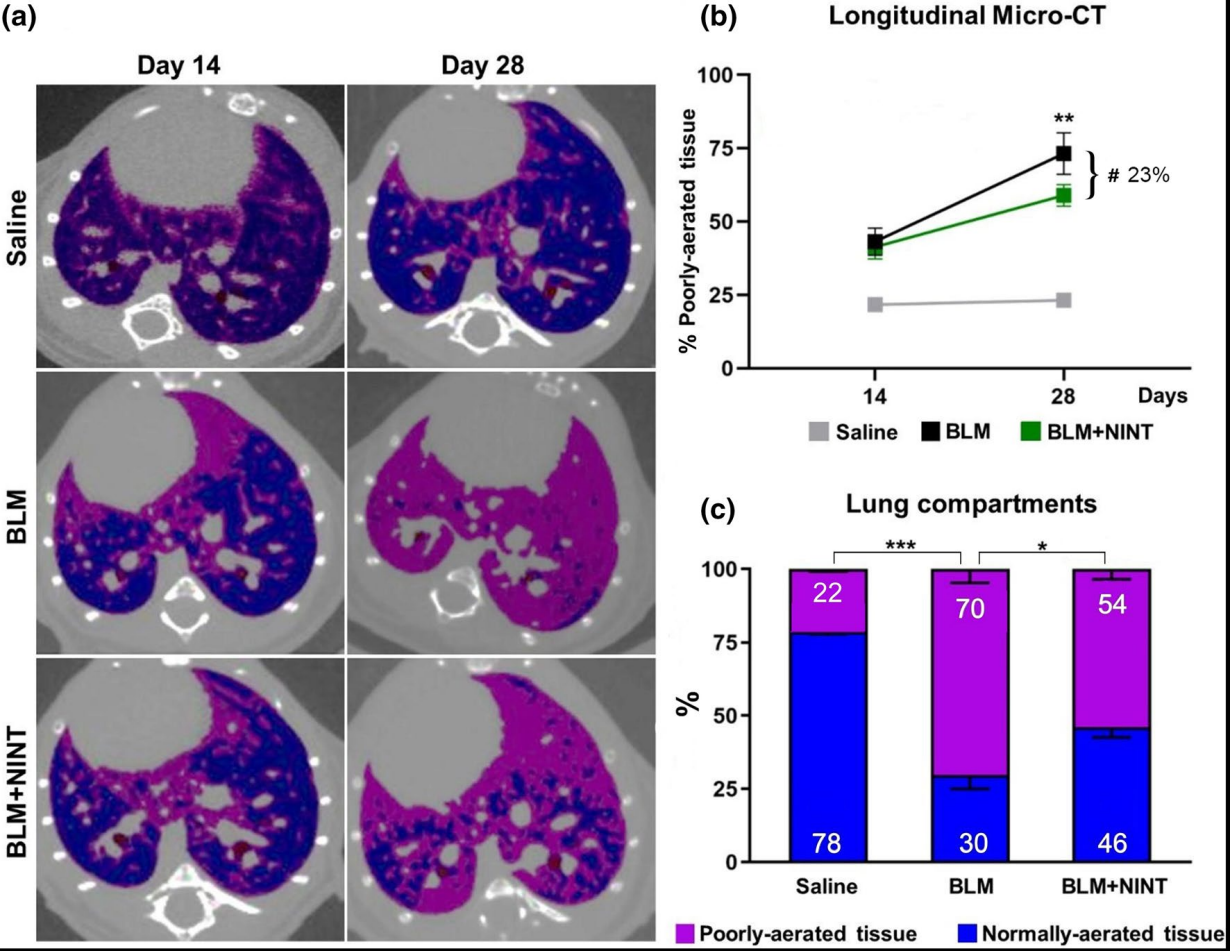
Longitudinal Micro-CT analysis. Representative Micro-CT images of Saline, BLM and BLM + NINT lungs showing normally and poorly- aerated tissue (colored in blue and pink, respectively) at day 14 and 28 (**a**). Longitudinal Micro-CT quantification of poorly-aerated tissue at day 14 and 28 in Saline, BLM and BLM + NINT groups (**b**). Asterisks indicate a significant increase in % poorly-aerated tissue from 14 to 28 days for the BLM group (**P < 0.01, two-way ANOVA followed by Sidak’s test). The inhibitory effect of NINT treatment has been calculated as reduction of poorly-aerated tissue in the BLM + NINT group with respect to BLM at day 28 (23% inhibition, ^#^ P < 0.05; two-way ANOVA followed by Dunnett’s test). Lung compartments quantification at day 28 in Saline, BLM and BLM + NINT group (**c**). Asterisks indicate statistical significance of both lung compartments comparing Saline and BLM + NINT groups with BLM as control (*P < 0.05, ***P < 0.001; Chi-squared test). Data are shown as mean percentages ± S.E.M. for 8 mice per group.

Representative micro-CT scans of BLM and BLM + NINT-treated mice showed an increase in the poorly-aerated tissue along with a decrease in the normo-aerated tissue (pink and blue, respectively) at 28 days compared to baseline (Fig. 6a). On the contrary, Saline lungs were largely composed of normally-aerated tissue and remained unchanged over time.

Poorly-aerated lung compartment was used as a marker of fibrosis, thus longitudinally quantified for each group (Fig. 6b). These data revealed that fibrosis was uniformly distributed at the baseline for BLM and BLM + NINT groups since the percentage of poorly-aerated tissue was not significantly different. Fibrosis progression was assessed in both BLM and BLM + NINT groups from day 14 to 28 but statistical significance was achieved only for the BLM group (Fig. 6b). NINT treatment significantly limited fibrosis progression (−23%) compared to vehicle-treated mice (Fig. 6b). Its antifibrotic effect was also evidenced by the quantification of normally and poorly-aerated lung compartments (Fig. 6c) at the end of pharmacological treatment: poorly-aerated tissue in BLM mice (70%) was significantly higher than in the Saline group (22%), whereas it was reduced in the NINT group (54%).

## Discussion

Despite the great advances in knowledge of the etiopathogenesis of SSc-ILD in recent years, medical need remains very high. In particular, it is imperative to find reproducible and relevant animal models capable of reproducing the chronic and progressive aspects of the disease, and to provide robust readouts in order to test putative new drugs^24,25^. Furthermore, new technologies need to be fully integrated into the antifibrotic drug development process for the screening of the best compounds to advance in clinical therapy.

The antifibrotic effects of NINT evaluated on lung and skin fibrosis in a SSc-ILD model, by using different readouts including longitudinal micro-CT imaging, will serve as a reference point for further preclinical studies.

In lung, NINT showed a significant inhibition of the total Ashcroft score and collagen deposition. Although the antifibrotic treatment effect was less pronounced on the Ashcroft frequency distribution, the number of fibrotic foci/mm^2^ and Σ AOI/frame (%); these parameters may still provide important information either to monitor the fibroproliferative alteration of lung parenchyma and the inter-experiment reproducibility of fibrosis, or to evaluate drug efficacy.

Even though Hyp is commonly considered an important readout, in our drug screening experiments concerning BLM-induced lung fibrosis models, we always observed high variability and poor inter-experiments reproducibility. As previously reported, the fibrotic lesions, mainly localized in the subpleural area, could be underestimated if evaluated over the whole parenchyma^15^. Furthermore, the quantification of Hyp could be affected by the size of the sampling site. For this reason, a FOI of 2 between Saline and BLM raises serious questions about whether this range will be sufficient to evaluate any antifibrotic effect in this model. Similar results on Hyp modulation have been reported in a BLM-induced lung fibrosis model^22^. Moreover, Hyp determination, being a destructive assay, precludes other histological analyses or alternative readouts that might be considered^33^.

NINT significantly inhibited WBC numbers in BALF, but only a moderate decrease was observed in macrophages, lymphocytes and neutrophils.

Although the pivotal role of macrophages in IPF has been recently well reported^34,35^, the modulatory effect of NINT on macrophage polarization in vivo has been only demonstrated in Fra2 transgenic mice, ameliorating histological features of pulmonary arterial hypertension^36^. In human macrophages, NINT treatment was able to downregulate M2 markers of expression *in vitro*^37^. Although NINT showed a modest inhibitory effect in our mouse model, we found that M2-like cells may represent a useful readout for evaluating the antifibrotic drug effect, since it might be directly linked to fibrosis.

In accordance with Ackermann et al. (2016)^22^ the decrease in inflammatory cells could explain the concomitant reduction of MMPs and their inhibitors. NINT reduced the levels of matrix metalloproteinases in BALF samples compared to vehicles, however only MMP-9 was significantly modulated since it is produced by alveolar inflammatory cells. MMP-2 and TIMP, instead, were not significantly reduced by NINT treatment probably because they are more expressed by structural cells (epithelial cells and interstitial macrophages respectively) which are less likely to be harvested by bronchoalveolar lavage^38,39^.

NINT treatment did not significantly reduce either the inflammatory infiltrate or lipoatrophy, which are the most evident changes induced by BLM administration through osmotic minipumps in the skin. This is in accordance with the evidence that, even though NINT has been approved for scleroderma, it has been shown not ameliorate the status of the skin^18,19^.

However, the effect of NINT has been reported in different skin fibrosis models based on multiple subcutaneous BLM injections and in different mouse strains^21^.

Unfortunately, many pre-clinical readouts focused on evaluating antifibrotic treatments are terminal procedures which don’t reflect the clinical situation. In this scenario, the inhibition of the drug group compared to vehicles remains the unique informative result, precluding any other intra-subject evaluation about the disease development.

Imaging technologies, such as micro-CT, allowed longitudinal studies in the same mice before (baseline) and after drug treatment as its own control^32^. This pre-clinical setting reflects a more relevant clinical situation along with a drastic reduction in both the variability and number of mice used.

The therapeutic protocol used for NINT, starting the treatment at day 14, could decrease the antifibrotic effect, as reported in the study, on different readouts; however, we are committed to bring out the real potentiality of the drug tested and no amplify the pharmacological activity.

NINT only partially reduced lung fibrosis progression in ILD patients^19^, since it has been reported a decline in the forced vital capacity (FVC) despite the treatment of 52 weeks. In agreement with the clinical outcome, the progressive increase in poorly-aerated tissue observed in BLM and NINT groups, compared to baseline, revealed a worsening of lung fibrosis which was only partially reduced by the antifibrotic treatment.

In this study we pharmacologically validated a new murine model of SSc-ILD, using an FDA-approved antifibrotic drug, and the effect of NINT was evaluated on both lung and skin. These findings highlight the reliability of this model, which could therefore serve for secondary screening to better profile new putative antifibrotic drugs.

## Supplementary Information

Supplementary Information 1.

Supplementary Information 2.

## Data Availability

All datasets generated for this study are included in the article/Supplementary Information.
