# Supplementary figures and images for: SSC-ILD mouse model induced by osmotic minipump delivered bleomycin: effect of Nintedanib
Source: Sci Rep. 2021 Sep 16;11:18513. doi: 10.1038/s41598-021-97728-z (PMC8445948; doi:10.1038/s41598-021-97728-z)

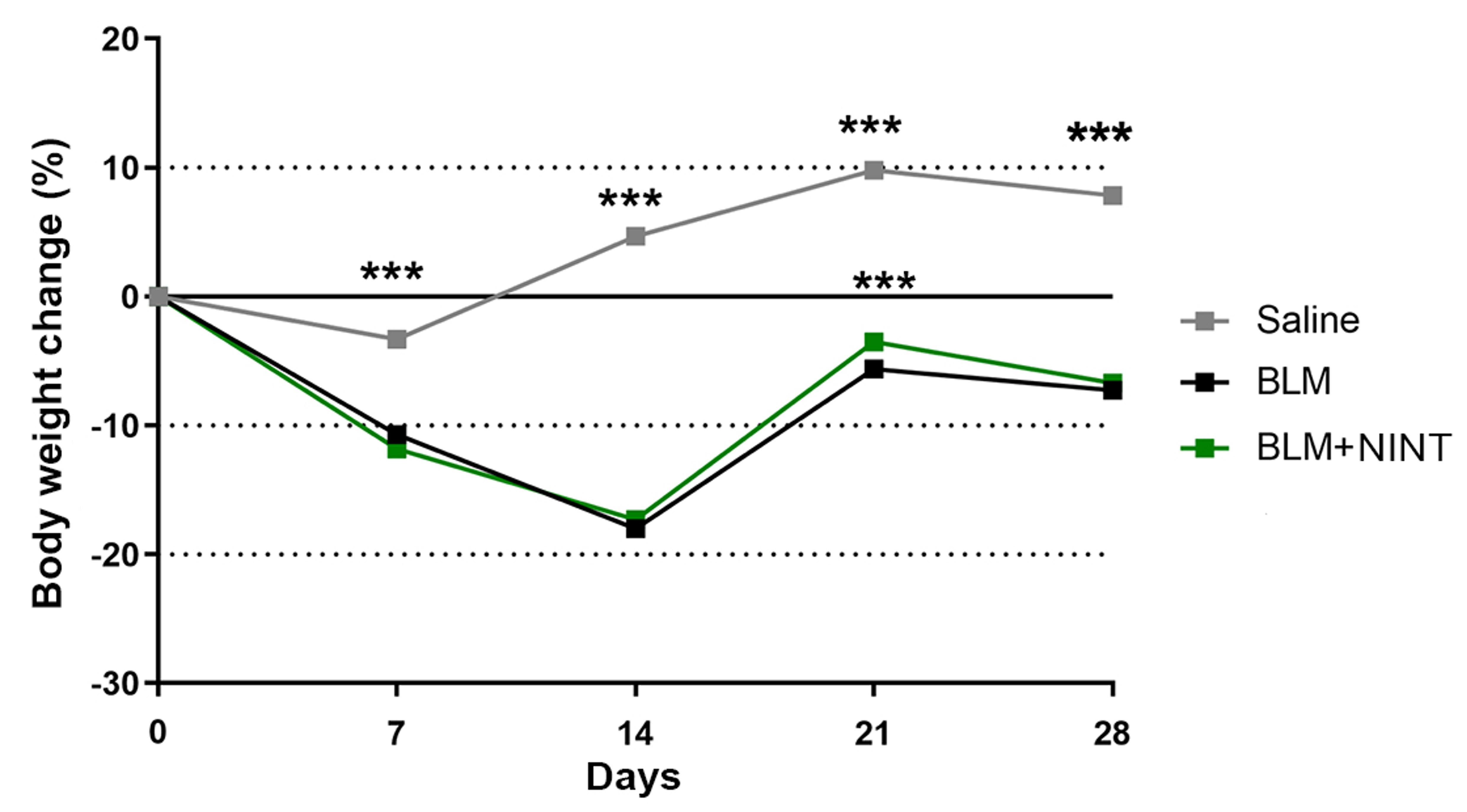

Supplement: Supplementary file 1 — Supplementary Information 1. [file 41598_2021_97728_MOESM1_ESM.jpg]

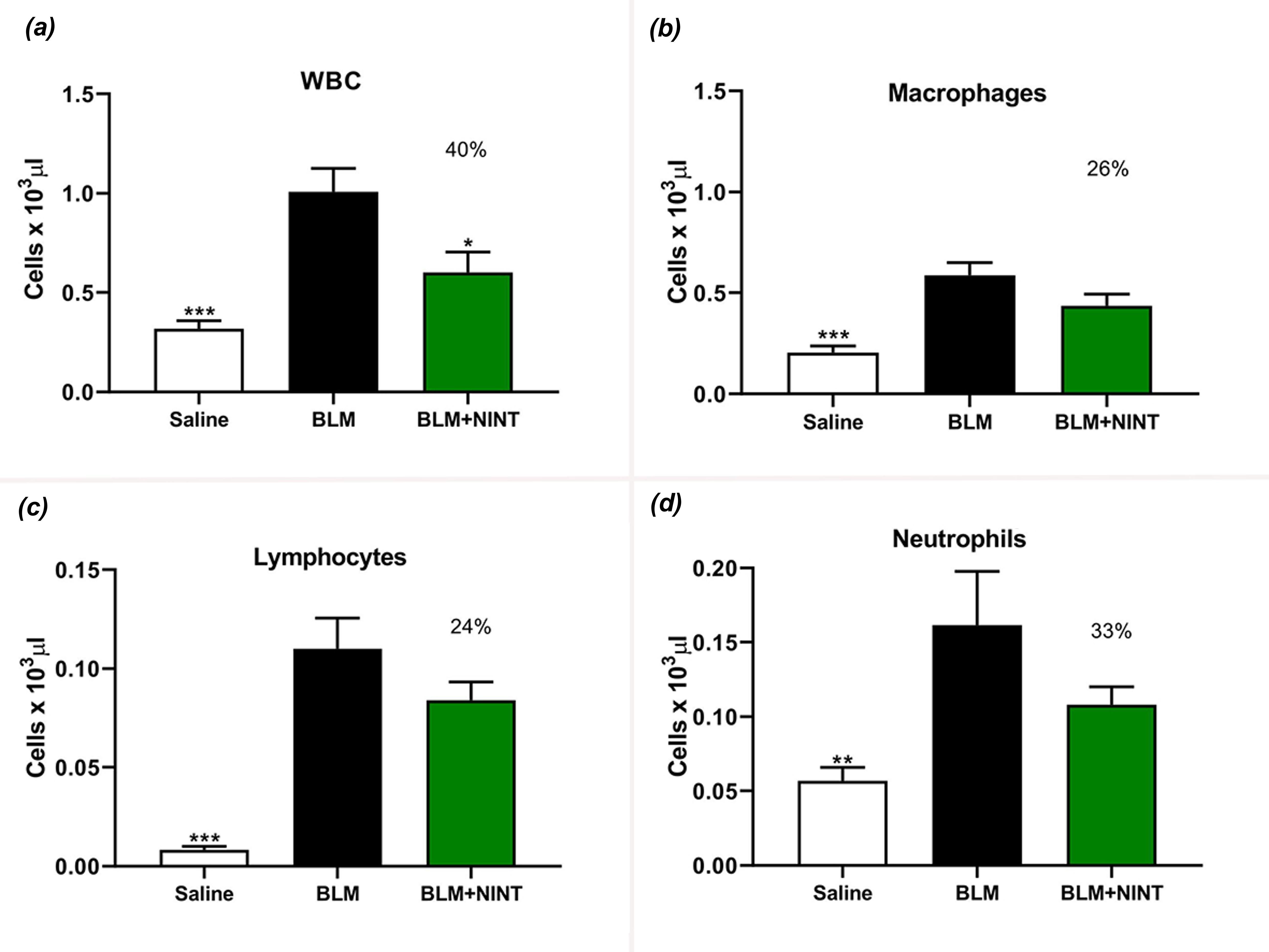

Supplement: Supplementary file 2 — Supplementary Information 2. [file 41598_2021_97728_MOESM2_ESM.jpg]
